# Supplementary material for: Association between oral microbiome and five types of respiratory infections: a two-sample Mendelian randomization study in east Asian population
Source: Front Microbiol. 2024 Apr 10;15:1392473. doi: 10.3389/fmicb.2024.1392473 (PMC11039966; doi:10.3389/fmicb.2024.1392473)
Supplement: Supplementary file 1 [file Data_Sheet_1.zip › Supplementary Materials/MR plots of saliva/Tonsillitis/s__Prevotella_nanceiensis_mgs_3029/leave_one_out.pdf]

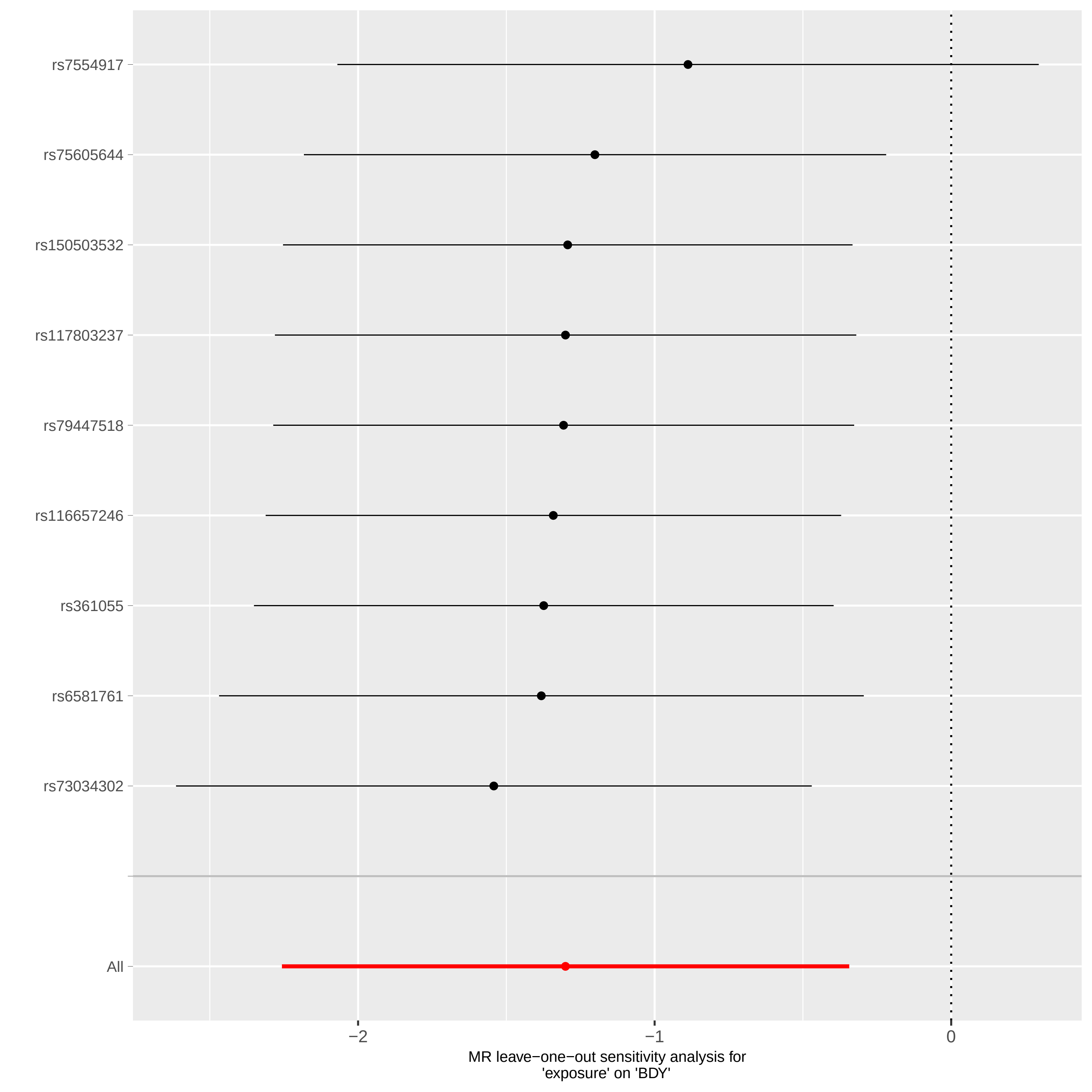
rs7554917
rs75605644
rs150503532
rs117803237
rs79447518
rs116657246
rs361055
rs6581761
rs73034302
All
−2
−1
0
MR leave−one−out sensitivity analysis for
'exposure' on 'BDY'
